# 

**Published:** 1895-07

**Authors:** 


### EDITORIAL.

### American Dental Association.

The thirty-fifth annual session of the American Dental Association will be held at Asbury Park, N. J., commencing Tuesday, August 6, 1895.

GEO. H. CUSHING, Rec. Sect'y.

THE following circular has just come to hand, and is fully explanatory of its intent. The object is one that in justice to the subject is an eminently worthy one. There is not a dentist in this country that is not indebted to Dr. Chapin A. Harris, and largely so, indeed more than can ever be paid; and it is to be hoped that every one will esteem it a great favor and indeed a pleasure to contribute to the object specified in this circular. And now we earnestly ask that every dentist and dental student will manifest their regard and love for the Father of Dentistry in this country by making a substantial contribution to the Harris Memorial Fund that a monument may be procured that will be a reminder not only to the present generation, but to many generations to come, of what Dr. Harris was and did for dentistry, not only in this but in all other civilized countries, for all are reaping the fruits of work he performed. Decide to contribute and decide to contribute at once, and decide to make the contribution one worthy the object.

THE annual meeeting of the Northern Iowa Dental Society will be held at Clear Lake, September 3d, 4th and 5th. A good program has been prepared and a very interesting and instructive meeting is expected. Thursday afternoon will be devoted to social enjoyment. A tour of the lake, regatta, etc. All dentists in Iowa and adjoining states are cordially invited.

For further information and programs, apply to Dr. G. H. Belding, Calmar, Iowa, or Dr. J. J. Crout, Rock Rapids, Iowa.

WM. H. STEELE, Chairman, Ex. Com.

### CONTENTS.

### COMMUNICATIONS.

### THE DENTAL REGISTER,

**A MONTHLY MAGAZINE DEVOTED TO THE INTERESTS OF THE**

**DENTAL PROFESSION.**

*Terms Reduced to* $2.00 *Per Year. Single Copies,* 25 *Cents.*

**EDITED BY PROF. J. TAFT, D.D.S.**

—AND—

**Published by SAM'L A. CROCKER & CO., Ohio Dental and Surgical Depot.**

The volume commences in January and closes in December.

The REGISTER being issued monthly is always fresh, therefore Indispensable to the practitioner who desires to keep posted up with the new inventions and improvements which are daily developed. Neither expense nor effort will be spared to give value and variety to its contents. Articles will be illustrated with well executed engravings whenever they will add to the interest or assist to explanation.

Its extensive circulation and frequency of issue make it one of the BEST DENTAL ADVERTISING MEDIUMS in the United States.

All subscriptions must begin with January or July.

Local or special notices, five lines or less, will be inserted for $3 each insertion ; over five lines, 50 cts. per line.

All advertising bills payable in advance, or at furthest, after the issue of the first number containing the advertisement. All transient advertisements to be paid for in advance.

☞CONTRIBUTIONS SOLICITED.

Exclusively Editorial Communications should be addressed to the Editor.

The latest number of the REGISTER may be ordered with or without other good at any time, and all letters should be addressed to

